# The Two β-Arrestins Regulate Distinct Metabolic Processes: Studies with Novel Mutant Mouse Models

**DOI:** 10.3390/ijms23010495

**Published:** 2022-01-02

**Authors:** Jürgen Wess

**Affiliations:** Molecular Signaling Section, Laboratory of Bioorganic Chemistry, National Institute of Diabetes and Digestive and Kidney Diseases, National Institutes of Health, Bethesda, MD 20892, USA; jurgenw@niddk.nih.gov; Tel.: +1-301-402-3589

**Keywords:** β-arrestins, G protein-coupled receptors, diabetes, obesity, metabolism, mutant mice

## Abstract

The two β-arrestins (β-arrestin-1 and -2; alternative names: arrestin-2 and -3, respectively) are well known for their ability to inhibit signaling via G protein-coupled receptors. However, β-arrestins can also act as signaling molecules in their own right. Although the two proteins share a high degree of sequence and structural homology, early studies with cultured cells indicated that β-arrestin-1 and -2 are not functionally redundant. Recently, the in vivo metabolic roles of the two β-arrestins have been studied using mutant mice selectively lacking either β-arrestin-1 or -2 in cell types that are of particular relevance for regulating glucose and energy homeostasis. These studies demonstrated that the β-arrestin-1 and -2 mutant mice displayed distinct metabolic phenotypes in vivo, providing further evidence for the functional heterogeneity of these two highly versatile signaling proteins.

## 1. Introduction

The two β-arrestins, β-arrestin-1 and -2 (alternative nomenclature: arrestin-2 and -3, respectively), are intracellular proteins that are best known for their ability to regulate the activity of G protein-coupled receptors (GPCRs) [1,2]. In contrast to rod and cone arrestin (arrestin-1 and -4, respectively), which are mainly found in the eye, β-arrestin-1 (βarr1) and β-arrestin-2 (βarr2) are found in virtually every cell type [1,2]. Following GPCR occupation by agonist ligands, including hormones, neurotransmitters, metabolites, or sensory stimuli, most GPCRs are subject to phosphorylation by GPCR kinases (GRKs). This structural modification enables the two β-arrestins to bind to the intracellular surface of the receptors, thus interfering with productive receptor/G protein coupling via steric hindrance [1,2]. Moreover, due to the ability of receptor-associated β-arrestins to bind to clathrin and adaptor protein 2 (AP-2), β-arrestins play a key role in GPCR internalization via clathrin-coated pits [2,3].

Beyond these “traditional roles” of β-arrestins, a large body of evidence indicates that β-arrestins can act as signaling molecules in their own right, often by serving as scaffolding proteins for various intracellular signal transduction cascades [4,5,6]. The best-known example is the ability of β-arrestins to stimulate signaling via different mitogen-activated protein kinase (MAPK) signaling pathways [4,5,6]. While many of these non-canonical β-arrestin activities are predicted to require the prior recruitment of β-arrestins by GPCRs, GPCR-independent β-arrestin functions have also been reported [4,5,6]. In addition, recent studies suggest that at least some of the cellular functions of β-arrestins require the presence of functional G proteins [7,8,9].

βarr1 and βarr2 are found in all mammals, suggesting that the existence of the two β-arrestin isoforms is advantageous from an evolutionary point of view [10]. The two β-arrestins share more than 70% identity at the amino acid level and have very similar three-dimensional structures [11]. For this reason, it is not surprising that the two proteins share many similar functions. However, early studies with cultured cells clearly indicated that βarr1 and βarr2 are not functionally redundant [1,11]. For example, βarr2 has higher affinity for many GPCRs than βarr1, although some GPCRs preferentially recruit βarr1 [10]. Another striking example highlighting this functional heterogeneity is the observation that βarr2, but not βarr1, can promote the activation of c-jun N-terminal kinase 3 (JNK3) [10]. In agreement with these findings, an early global proteomics study using cultured HEK293 cells showed that the two β-arrestins are endowed with distinct protein interaction profiles, both under basal conditions and after stimulation of angiotensin II type 1a receptor signaling [12].

One possibility is that minor local conformational differences between βarr1 and βarr2 contribute to the ability of the two proteins to affect cellular functions in an isoform-specific fashion. In agreement with this notion, subtle structural differences have been observed in the inter-domain hinge region of activated βarr1 and βarr2 [11].

While the two β-arrestins are primarily found in the cytoplasm, both βarr1 and βarr2 contain a nuclear localization sequence [13]. However, since βarr2 also harbors a nuclear export signal domain, βarr2, but not βarr1, is predicted to be rapidly exported back to the cytoplasm [11]. In agreement with this concept, accumulating evidence indicates that nuclear βarr1 can regulate several important transcriptional processes [13]. These findings suggest that differences in subcellular localization can also contribute to the functional divergence of the two β-arrestins [11].

## 2. Studies with Whole-Body β-Arrestin Knockout (KO) Mice

In agreement with published in vitro data, studies with whole-body β-arrestin KO mice confirmed that βarr1 and βarr2 differ in the physiological processes they regulate in vivo [14]. For example, nicotinic acid (niacin), an FDA-approved drug, lowers the plasma lipid levels by activation of the G_i_-coupled hydrocarboxylic acid 2 (HCA_2_) receptors (alternative name: GPR109A) expressed by adipocytes [15]. A major side effect caused by nicotinic acid is the “niacin flush”, a flush of red on the skin that is frequently accompanied by an itching or burning sensation. This response is greatly reduced in βarr1 KO but not βarr2 KO mice [16]. Another striking example highlighting the different in vivo functions of the two β-arrestins are the different metabolic phenotypes displayed by whole-body βarr1 and βarr2 KO mice [17].

## 3. Analysis of Cell-Type Specific β-Arrestin KO Mice

The recent availability of floxed βarr1 and βarr2 mice has made it possible to delete either of the two β-arrestin isoforms in a cell type-specific fashion [18,19]. As a result, it is now possible to compare the in vivo physiological importance of βarr1 and βarr2 expressed by a particular cell type. Specifically, recent work has targeted cell types that play critical roles in regulating glucose and energy homeostasis [20]. In the following, I will briefly summarize the outcome of studies carried out with cell type-specific β-arrestin KO mice that provide additional in vivo evidence for the functional heterogeneity of the two β-arrestin isoforms.

### 3.1. Hepatocytes

**Figure 1:**
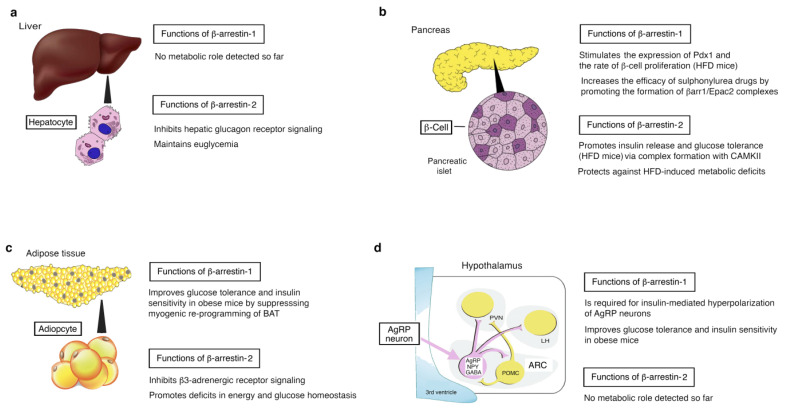
The two β-arrestins regulate different functions in metabolically important cell types in vivo. (**a–d**) Summary of the outcome of metabolic studies with mutant mice lacking βarr1 or βarr2 selectively in mouse hepatocytes (**a**), pancreatic β-cells (**b**), adipocytes (**c**), and AgRP neurons of the arcuate nucleus of the hypothalamus (**d**) (for a review, see [20]). See text for details. HFD, high-fat diet; CAMKII, calcium/calmodulin-dependent protein kinase II; BAT, brown adipose tissue; AgRP, agouti-related peptide; NPY, neuropeptide Y; POMC, proopiomelanocortin; ARC, arcuate nucleus; PVN, paraventricular nucleus; LH, lateral hypothalamus.

Mice that selectively lack βarr1 in hepatocytes do not show any impairments in glucose homeostasis [21]. In contrast, hepatocyte-specific βarr2 KO mice display striking metabolic deficits, primarily due to increased hepatic glucagon receptor (GCGR) signaling [21] (Figure 1a). While glucagon-induced GCGR internalization remains intact in hepatocytes lacking βarr1, this process is severely disrupted in βarr2-deficient hepatocytes. Since receptor internalization contributes to GPCR desensitization, the most likely scenario is that the lack of GCGR internalization caused by βarr2 deficiency plays a key role in promoting GCGR signaling in βarr2-deficient hepatocytes [21].

### 3.2. Pancreatic β-Cells

The selective inactivation of βarr1 or βarr2 in pancreatic β-cells also results in well-defined metabolic phenotypes [22,23,24] (Figure 1b). Mice that selectively lack β-cell βarr2 show significantly impaired insulin release when the mutant mice are maintained on a calorie-rich diet [23]. Studies with isolated islets showed that glucose-induced insulin secretion is greatly reduced in βarr2-deficient β-cells, most likely due to impaired function of calcium/calmodulin-dependent protein kinase type II (CAMKII), a multi-functional Ser/Thr protein kinase that plays an important role in promoting insulin exocytosis [23]. Biochemical studies indicated that βarr2 can interact with CAMKII in a protein complex that is critical for the proper function of CAMKII [23]. It remains unknown at present whether this βarr2 function is regulated by the activity of β-cell GPCRs.

Like the β-cell βarr2 KO mice, β-cell βarr1 KO mice display significant impairments in glucose tolerance and glucose-dependent insulin secretion when maintained on an obesogenic diet [24]. Interestingly, Barella et al. [24] reported that obese β-cell βarr1 KO mice exhibit a striking decrease in β-cell mass and rate of β-cell proliferation (Figure 1b). Additional studies showed that β-cell βarr1 is required for the proper expression of the transcription factor Pdx1, the master regulator of β-cell function and β-cell mass expansion [25]. Barella et al. [24] concluded that the lack of nuclear βarr1 leads to reduced Pdx1 expression and that this deficit underlies the metabolic impairments displayed by obese β-cell βarr1 KO mice [24]. 

Somewhat surprisingly, a related study [22] showed that the presence of β-cell βarr1 is required for most antidiabetic drugs of the sulfonylurea (SU) family to simulate insulin release with high efficacy (Figure 1b). Mechanistic studies revealed that βarr1 enhances SU-induced insulin release by promoting SU-dependent activation of Epac2 via formation of a βarr1/Epac2 complex that triggers Rap1 activation and insulin secretion [22]. 

### 3.3. Adipocytes

Mice that selectively lack βarr2 in adipocytes are protected against high-fat diet-induced weight gain and the associated metabolic deficits, including impaired glucose tolerance and insulin resistance [26]. Pydi et al. [26] showed that the metabolic improvements caused by adipocyte βarr2 deficiency are mediated by the browning/beiging of white adipose tissue. At the cellular level, βarr2 acts as a strong negative regulator of adipocyte β3-adrenergic receptor (β3-AR) signaling by promoting the internalization of this receptor subtype [26] (Figure 1c). In mice, β3-ARs are known to mediate the browning/beiging of white adipose tissue caused by activation of the sympathetic nervous system [27].

Strikingly, mutant mice that selectively lack βarr1 in adipocytes exhibit metabolic phenotypes that are opposite to those caused by adipocyte βarr2 deficiency [28]. The absence of βarr1 in adipocytes results in greatly impaired glucose tolerance and insulin resistance when mice are maintained on an obesogenic diet. Pydi et al. [28] demonstrated that βarr1 deficiency promotes the expression of myostatin in brown adipose tissue, resulting in elevated plasma myostatin levels that eventually trigger peripheral insulin resistance. These and other findings indicate that βarr1-mediated suppression of myostatin expression by brown adipose tissue is required for maintaining proper insulin responsiveness and blood glucose homeostasis [28] (Figure 1c).

### 3.4. Agouti-Related Protein (AgRP) Neurons 

AgRP neurons, located in the arcuate nucleus of the hypothalamus, play a key role in the central regulation of food intake, energy expenditure, and glucose homeostasis [29]. Interestingly, mutant mice selectively lacking βarr1 in AgRP neurons mice show significant impairments in glucose tolerance and insulin sensitivity when consuming an obesogenic diet [30] (Figure 1d). This phenotype was not observed with mice in which βarr2 was selectively inactivated in AgRP neurons [30]. Electrophysiological studies indicated that βarr1 is required for the ability of insulin to ‘silence’ AgRP neurons, resulting in multiple beneficial metabolic effects. One possible mechanism underlying this finding is the ability of βarr1 to stabilize insulin receptor substrate 1 (IRS-1), a key transducer of insulin receptor signaling, via complex formation [30]. 

### 3.5. Concluding Remarks

In summary, studies with cell type-specific βarr1 and βarr2 mutant mice strongly support the concept that the two β-arrestins regulate distinct physiological processes. While some of these effects can be explained by the traditional roles of β-arrestins as inhibitors of GPCR signaling, many of the phenotypes observed with the newly developed β-arrestin mutant mice are consistent with alternative β-arrestin functions. It remains to be determined to which extent these novel β-arrestin functions are regulated by GPCR signaling and/or GPCR/β-arrestin interactions. The outcome of the phenotyping studies summarized in this short article may guide the development of novel drugs capable of modulating the βarr1 or βarr2 activity or expression levels for the treatment of various pathophysiological conditions, including type 2 diabetes and related metabolic disorders (for a detailed review of potential therapeutic opportunities, see [20]).
